# Dynamic Causal Modeling and Online Collaborative Forecasting of Air Quality in Hong Kong and Macao

**DOI:** 10.3390/e25091337

**Published:** 2023-09-15

**Authors:** Cheng He, Jia Ren, Wenjian Liu

**Affiliations:** 1 Faculty of Data Science, City University of Macau, Macao 999078, China; d21092100101@cityu.mo (C.H.); andylau@cityu.mo (W.L.); 2 Industrial Design School, Shandong University of Art & Design, Jinan 250399, China; 3 School of Information and Communication Engineering, Hainan University, Haikou 570208, China

**Keywords:** explainable artificial intelligence, machine learning, dynamic Bayesian network, causality, collaborative forecast

## Abstract

The Hong Kong and Macao Special Administrative Regions, situated within China’s Guangdong–Hong Kong–Macao Greater Bay Area, significantly influence and are impacted by their air quality conditions. Rapid urbanization, high population density, and air pollution from diverse factors present challenges, making the health of the atmospheric environment in these regions a research focal point. This study offers three key contributions: (1) It applied an interpretable dynamic Bayesian network (DBN) to construct a dynamic causal model of air quality in Hong Kong and Macao, amidst complex, unstable, multi-dimensional, and uncertain factors over time. (2) It investigated the dynamic interaction between meteorology and air quality sub-networks, and both qualitatively and quantitatively identified, evaluated, and understood the causal relationships between air pollutants and their determinants. (3) It facilitated an online collaborative forecast of air pollutant concentrations, enabling pollution warnings. The findings proposed that a DBN-based dynamic causal model can effectively explain and manage complex atmospheric environmental systems in Hong Kong and Macao. This method offers crucial insights for decision-making and the management of atmospheric environments not only in these regions but also for neighboring cities and regions with similar geographical contexts.

## 1. Introduction

The Guangdong–Hong Kong–Macao Greater Bay Area, one of China’s most economically robust and dynamic regions, is experiencing rapid urbanization [1]. Nonetheless, this swift progress is concurrently leading to a deterioration in air quality. The atmospheric environmental conditions in this region significantly influence the air quality of the Hong Kong and Macao Special Administrative Regions, demonstrating high sensitivity [2,3]. Situated in the subtropical monsoon climate zone, Hong Kong spans approximately 1114 square kilometers and is home to around 7.33 million people. In contrast, Macao, with a population of nearly 670,000, covers a mere 33 square kilometers. Hong Kong, in particular, is grappling with escalating levels of nitrogen dioxide (NO_2_) pollution, which is noted for its deleterious effects on the human respiratory system. This pollutant can undermine the body’s resistance to diseases, exacerbating the condition of individuals with chronic respiratory issues. In terms of NO_2_, the Causeway Bay Roadside Monitoring Station has recorded both the highest 1 h average (301 μg/m^3^) and the highest annual average (71 μg/m^3^), which serve as key Air Quality Indicators. Meanwhile, Macao faces a significant challenge with ozone (O_3_) pollution. O_3_ can induce chronic or acute impacts on human lung function, causing respiratory issues, diminished lung function, weakened immune system capabilities, and cardiovascular problems. Notably, the Taipa High Density Residential Area Station has recorded the most substantial annual average increase in O_3_ concentration, followed by the Taipa General Station. The air quality in both Hong Kong and Macao is a function of a myriad of complex factors.

The air quality of Hong Kong and Macao is influenced by several key parameters. Firstly, their coastal locations along the South China Sea, situated at the confluence of the tropics and subtropics, render them susceptible to climatic conditions stemming from both the Pacific Ocean and mainland China [4]. Secondly, their unique topographical features, characterized by abundant hills, valleys, and coastlines in Hong Kong and primarily plains and hills with low-lying urban areas in Macao, impact the wind flow and distribution of air pollutants. Moreover, air quality is also affected by variations in air pressure, temperature, and humidity [5,6]. Elevated air pressure leads to air stability, which can trap pollutants, while lower air pressure can cause air instability, facilitating the dispersal of pollutants. Furthermore, high temperatures and humidity can amplify surface-level photochemical reactions, promoting the formation of O_3_ [7]. Simultaneously, the copious sunshine in both regions, paired with potent sunlight, intensifies photochemical reactions, thereby affecting air quality. Lastly, wind patterns significantly influence air quality. Hong Kong and Macao encounter the monsoon influence persistently throughout the year. This meteorological phenomenon consists of two distinct phases: the summer monsoon (southwest monsoon) and the winter monsoon (northern monsoon). From April to September annually, the moist summer monsoon originating from the ocean leads to recurrent rainfall. Consequently, this phenomenon exhibits the potential to enhance air quality. Conversely, from October to March annually, the arid winter monsoon originating from the mainland transports transboundary air pollutants originating from the Pearl River Delta Industrial Zone. Urbanization, high population density, industrial activities, energy consumption, traffic congestion, and dense construction significantly strain the air quality in Hong Kong and Macao. The air quality in Hong Kong and Macao is subject to a range of influencing processes, encompassing natural phenomena, climatic and meteorological dynamics, atmospheric diffusion mechanisms, and chemical reactions. Therefore, decision-making and management concerning the atmospheric environment in these regions carry immense significance for air quality improvement, atmospheric environmental protection, and sustainable development. However, this atmospheric environmental decision-making is inherently complex and multifaceted, necessitating an integrated approach that considers a broad spectrum of parameters and processes impacting air quality.

Liu et al. (2018) employed the ARIMAX research approach, amalgamating the autoregressive integrated moving average (ARIMA) technique with numerical prediction, to formulate a predictive model for Hong Kong’s air quality [8]. They proposed a hybrid predictive strategy that facilitated dynamic real-time prediction. Nevertheless, ARIMAX rests on assumptions of linear trends and seasonality, and it inadequately articulates the causal interplay among variables, leading to frailty in model interpretation. Lei et al. (2020) harnessed the methodologies of multiple linear regression (MLR) and classification regression tree (CART) to establish an air quality prediction model for Macao [9]. They validated this model concerning instances of elevated and diminished pollution occurrences in 2019, encompassing the period coinciding with the outbreak of novel coronavirus pneumonia. Mendes et al. (2022) employed multiple regression (MR) and CART techniques to fabricate a predictive framework for air quality in Portugal and Macao [10]. This framework successfully anticipated pollution events, thus serving as a robust mechanism for safeguarding public health. However, MLR and MR assume linear associations within data, rendering them incapable of capturing intricate nonlinear relationships. Furthermore, these methods are susceptible to the influences of outliers and multicollinearity, consequently impinging on their predictive accuracy. Although CART can execute both classification and regression tasks, it lacks the capacity to offer probabilistic explanations, thereby constricting its range of applications. Moreover, the inflexible structure of decision trees within the CART model presents challenges in accommodating evolving information. Lei et al. (2023) adopted a diverse spectrum of research methodologies, encompassing artificial neural network (ANN), random forest (RF), extreme gradient boosting (XGBoost), support vector machine (SVM), and MLR, to fashion a predictive model for Macao’s air quality [11]. Their model successfully anticipated air pollutant concentrations over 24 h and 48 h horizons. However, it should be noted that ANN and XGBoost are susceptible to overfitting when dealing with intricate models, thereby compromising their generalizability. Furthermore, the computationally demanding nature of RF poses a challenge. SVM, while proficient in prediction, lacks the ability to furnish probabilistic explanations, thus circumscribing its scope of application and its intuitive interpretation. This study introduced the interpretable dynamic Bayesian network (DBN) research approach to forge a dynamic causal model for air quality in both Hong Kong and Macao.

The DBN constitutes an extension of the Bayesian network (BN) and is developed on the foundation of the Bayesian theorem, integrating principles from probability theory and graph theory, known as the probability graph model [12,13]. The DBN proves adept at amalgamating diverse time series data types originating from heterogeneous data sources. It excels in both modeling and reasoning about time-varying stochastic processes [14,15,16]. In the realm of atmospheric environment research, DBN stands as the principal modeling framework and an effective approach for addressing intricate atmospheric environmental decision-making and management quandaries. In the face of comprehensive modeling challenges presented by atmospheric environment systems, which encompass time series, multi-dimensionality, instability, and uncertainty, DBN circumvents the need for presumptions regarding data distribution and stationarity. It materializes expeditiously through an intuitively structured graphical framework, enabling the exploration of potential interplays among distinct variables. The causal relationships between nodes are estimated through conditional probabilities. DBN amalgamates numerous advantages. Primarily, it adeptly captures intricate nonlinear relationships and interaction effects, thus transcending the limitations inherent in linear models. Secondly, DBN adeptly manages uncertainty, demonstrating its prowess in handling outliers and noisy data. Consequently, it effectively mitigates the impact of multicollinearity. Moreover, DBN enriches model interpretability by offering a probabilistic framework that distinctly delineates causal relationships between variables. Simultaneously, DBN accommodates modular modeling with enhanced flexibility, thereby manifesting robust generalization capabilities. Lastly, DBN integrates prior knowledge, dynamically selects and adjusts parameters, and achieves computational efficiency. Therefore, DBN emerges as a pivotal instrument for crafting a dynamic causal model delineating air quality within Hong Kong and Macao. Its utility lies in its capacity to elucidate and manage the intricate atmospheric environment system, thereby supporting decision-making and management endeavors pertaining to the atmospheric environment in both regions.

This study offers three key contributions: (1) It applied an interpretable DBN to construct a dynamic causal model of air quality in Hong Kong and Macao, amidst complex, unstable, multi-dimensional, and uncertain factors over time. (2) It investigated the dynamic interaction between meteorology and air quality sub-networks, and both qualitatively and quantitatively identified, evaluated, and understood the causal relationships between air pollutants and their determinants. (3) It facilitated an online collaborative forecast of air pollutant concentrations, enabling pollution warnings. The insights gained from this study may serve as a valuable reference for neighboring cities and regions sharing similar geographical contexts with Hong Kong and Macao. The paper is structured as follows: Section 2 introduces the study area and data sources, DBN, DBN modeling process, DBN evaluation and validation; Section 3 discusses the results of DBN modeling and prediction; Section 4 concludes this work.

## 2. Materials and Methods

### 2.1. Study Area and Data Sources

Situated on the southeastern coast of China, the Hong Kong Special Administrative Region lies on the eastern bank of the Pearl River Estuary, across the sea to the west from its counterpart, Macao. Encompassing an area of approximately 1114 square kilometers, Hong Kong comprises Hong Kong Island, the Kowloon Peninsula, the New Territories, and an additional 262 islands. Home to a population of about 7.33 million, it boasts a per capita GDP of USD49,800. Conversely, the Macao Special Administrative Region also resides on China’s southeastern coast, positioned on the western bank of the Pearl River Estuary, with the sea separating it from Hong Kong to the east. Macao, with an area of about 33 square kilometers, comprises the Macao Peninsula, Taipa Island, Coloane Island, and Cotai City. It sustains a population of approximately 670,000 and yields a per capita GDP of USD43,900. Both Hong Kong and Macao are nestled within the subtropical region and experience a marine monsoon climate characterized by high heat, ample water vapor, elevated temperatures, and frequent rain. The annual average temperature in Hong Kong is 23.9 °C, accompanied by an annual precipitation of 2205.4 mm. Macao, in contrast, maintains an average annual temperature of 22.7 °C and sees an annual precipitation of 2030.8 mm. The typhoon season in both Hong Kong and Macao typically extends from June to October.

Hong Kong’s air quality monitoring network, consisting of 18 stations—15 general and 3 roadside—tracks the concentrations of major air pollutants. The primary source of coarse particulate matter (PM_10_) and fine particulate matter (PM_2.5_) emissions in Hong Kong originates from combustion processes. They encompass a range of sources, namely traffic and marine emissions, industrial and power generation activities, culinary processes, as well as biomass and open burning. PM_10_, associated with chronic or acute effects on lung function, can instigate respiratory problems. However, due to its diminutive size, PM_2.5_, capable of penetrating deep into the lungs, poses a more severe impact on human health. The highest annual average concentration of PM_10_ was registered at the Causeway Bay Roadside Monitoring Station, whereas the highest 24 h average for PM_2.5_ was recorded at the Yuen Long General Monitoring Station, and its highest annual average was noted at the Causeway Bay roadside station. Additionally, vehicular exhaust constitutes the principal source of carbon monoxide (CO) emissions in Hong Kong. CO, upon entering human blood vessels, may lessen the oxygen supply to various organs and tissues, resulting in symptoms akin to poisoning such as dyspnea, chest pain, headaches, and coordination loss—posing a heightened risk to individuals with heart diseases. The highest 8 h average of CO was detected at the Central Roadside Monitoring Station. Similarly, combustion processes are the leading sources of NO_2_ emissions in the city. NO_2_ is known to diminish the human respiratory system’s resilience to diseases and exacerbate the condition of those with chronic respiratory illnesses. The Causeway Bay Roadside Monitoring Station reported the highest 1 h average (301 μg/m^3^) and annual average (71 μg/m^3^) for NO_2_. Notably, all three roadside monitoring stations failed to meet the 1 h and annual air quality targets for NO_2_.

Macao’s air quality monitoring network, encompassing 6 stations, assesses the concentrations of primary air pollutants. The construction industry and land transportation contribute significantly to the emission of PM_10_ and PM_2.5_ in Macao, accounting for approximately 40% and 25% of these particulates, respectively. The annual average concentration of PM_10_ at the Taipa High Density Residential Area Station is measured at 52.3 (μg/m^3^), surpassing the established standard of 50 (μg/m^3^). Meanwhile, the annual average concentration of PM_2.5_ at the Taipa General Station is measured at 14.9 (μg/m^3^), reflecting a year-on-year increase of 9.6%. Land transportation predominates as the principal source of CO emissions in Macao. Notably, O_3_ pollution remains significant in Macao, broadly impacting air quality within the Guangdong–Hong Kong–Macao Greater Bay Area. O_3_ is generated via a photochemical reaction involving oxygen (O_2_), nitrogen oxides (NOx), and volatile organic compounds (VOC) under sunlight. Controlling VOC emissions emerges as a critical approach to managing O_3_ and photochemical pollution. The primary emission sources of non-methane volatile organic compounds (NMVOC) in Macao include organic solvents, land transportation, fuel supply, marine transportation, and sewage treatment. O_3_ can exert chronic or acute effects on human lung function, leading to breathing problems, diminished lung functionality, reduced immune system function, and cardiovascular issues. The annual average concentration of O_3_ at the Taipa High Density Residential Area Station is measured at 42.7 (μg/m^3^), reflecting a year-on-year increase of 25.2%. Meanwhile, the annual average concentration at the Taipa General Station is measured at 63.9 (μg/m^3^), reflecting a year-on-year increase of 5.8%.

**Figure 1:**
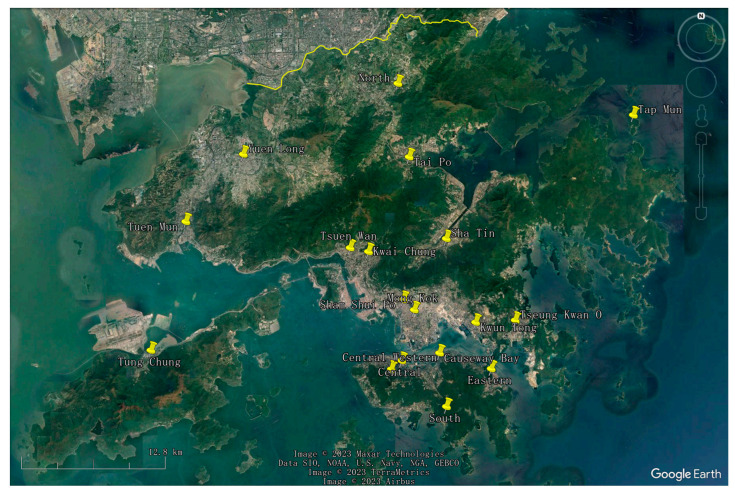
Hong Kong air quality monitoring network.

**Figure 2:**
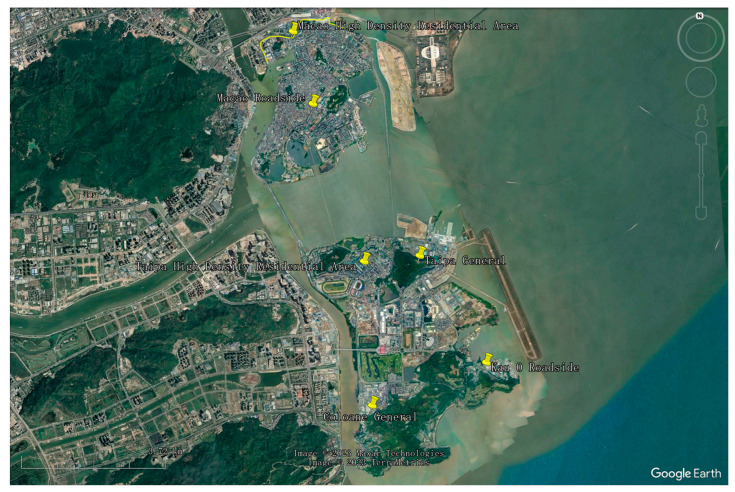
Macao air quality monitoring network.

The air quality in Hong Kong and Macao is influenced by a multitude of complex factors. Firstly, their geographical positioning at the junction of the tropics and subtropics, bordering the Asian continent to the north and the tropical ocean to the south, exposes them to both mid- and high-latitude atmospheric circulation from the mainland as well as low-latitude circulation from the ocean. This distinct geographical location results in conspicuous winter–summer circulation transitions, characterizing these regions as typical monsoon climate zones. Secondly, topographical features such as hills, valleys, and coastlines in Hong Kong, and plains, hills, and the predominantly low and flat urban areas in Macao, affect wind flow patterns and the distribution of air pollutants. For instance, valleys can generate a “tailpipe” effect, concentrating vehicle emissions and amplifying air pollution in specific areas. Furthermore, fluctuations in air pressure, temperature, and humidity also significantly impact air quality. High air pressure stabilizes the air, allowing pollutants to linger, whereas low air pressure induces instability, enabling the dispersion of pollutants. Concurrently, high temperature and humidity can augment surface photochemical reactions, fostering the formation of O_3_. Hong Kong and Macao, regions abundant in sunshine, experience intensified photochemical reactions due to strong sunlight, further affecting air quality. Wind patterns also affect air quality, with the winter monsoon importing transboundary air pollutants from the Pearl River Delta industrial zone, while the summer monsoon, bringing frequent rainfall, aids in enhancing air quality. Lastly, factors like urbanization, population density, industrial and energy consumption, traffic congestion, and extensive construction activities exert substantial pressure on the air quality of both Hong Kong and Macao. The Hong Kong air quality monitoring network is shown in Figure 1. The Macao air quality monitoring network is shown in Figure 2.

The present study utilized a composite dataset comprising meteorological parameters, air quality indices, and ancillary data, gathered from Hong Kong and Macao between 2016 and 2022. Meteorological measures from both regions encompass mean sea level pressure (MSLP)(hPa), highest, mean, and lowest temperatures (HT, MT, LT)(°C), dew point temperature (Td)(°C), relative humidity (RH)(%), average wind speed (AWS)(km/h), and precipitation (PR)(mm). In terms of air quality, Hong Kong’s dataset includes average concentrations of PM_10_, PM_2.5_, CO, and NO_2_ (μg/m^3^), whereas Macao’s dataset features average concentrations of PM_10_, PM_2.5_, CO, and O_3_ (μg/m^3^). Other data from both regions incorporate the sunshine duration (SSI)(h). In sum, this collective data pool provided a comprehensive resource for a granular understanding of the regional atmospheric conditions over the period of interest.

The data for Hong Kong were sourced from the Hong Kong Observatory and the Hong Kong Environmental Protection Department. The latter included readings from the Central Roadside Monitoring Station, the Yuen Long General Monitoring Station, and the Causeway Bay Roadside Monitoring Station. Correspondingly, the Macao dataset was obtained from the Macao Meteorological and Geophysical Bureau and the Macao Environmental Protection Bureau, which involved measurements from the Macao High Density Residential Area Station, the Taipa General Station, and the Coloane General Station. The data type and source and variable selection and description is shown in Table 1.

### 2.2. Dynamic Bayesian Network

BN is formulated on the foundations of the Bayesian theorem, intricately integrating probability theory and the graphical model theory of probability [17,18]. DBN, a derivative of BN, is a probabilistic graphical model specifically tailored to process time series data [19]. The term ‘dynamic’ in this context refers to a temporal sequence or process where interdependencies among variables may extend across varying time steps, providing a powerful tool to model and make sense of random processes that unfold over time. The fundamental architecture of DBN comprises two structures: the internal structure and the transfer structure [20]. The internal structure presupposes the existence of a BN at every ‘time slice’, succinctly describing the probabilistic interrelationships among all the random variables present at that point in time. On the other hand, the transfer structure encapsulates the modelling of transitions from one time slice to the subsequent one, elucidating the temporal evolution of variables. DBN inherently possess a Markovian property, implying that the current state, given a preceding state, is effectively independent of all past states [21]. The learning methodology within DBN is bifurcated into structure learning and parameter learning. While structural learning aims at establishing causal linkages among variables, parametric learning is responsible for discerning the probabilistic relationships among them. Given their capabilities, DBNs serve as vital theoretical apparatuses for the representation and reasoning of uncertain knowledge.

DBNs offer significant advantages in tackling the timing, multi-dimensionality, instability, and uncertainty associated with the atmospheric environment system, facilitating both systematic visualization and quantification [22,23]. Firstly, DBNs provide a comprehensive framework to comprehend and elucidate intricate atmospheric environmental systems in atmospheric modeling. These systems are composed of multiple, interrelated subsystems, each with its unique spatio-temporal dynamics. DBNs empower us to formulate and test hypotheses addressing these complex interactions, fostering a profound understanding of atmospheric environmental systems. Secondly, DBNs present an efficacious strategy to grapple with the uncertainties inherent in atmospheric environmental data [24]. Such data are frequently influenced by a myriad of factors, including measurement errors, model inaccuracies, and natural variability. The robustness of the model is enhanced by DBNs through the incorporation of prior probabilities and the application of Bayesian inference to accommodate these uncertainties. Lastly, DBNs also underpin decision-making processes related to the atmospheric environment. They are capable of predicting and quantifying the potential ramifications of specific decisions. Consequently, DBNs emerge as indispensable tools for air-quality modeling and analysis. They not only aid in demystifying the complexity of atmospheric environmental systems but also support decision-making management in the atmospheric environment.

### 2.3. Dynamic Bayesian Network Modeling Process

During the development of DBN models, the role of data preprocessing is paramount. Regarded as the preliminary phase of data analysis, the purpose of data preprocessing is to convert raw data into a format apt for subsequent analysis. The primary responsibilities encompassed by data preprocessing consist of handling missing data and performing data discretization.

Handling missing data is an essential aspect of data preprocessing. Missing data is a prevalent issue in real-world data collection, with potential causes ranging from equipment failure and lack of information to incomplete data acquisition. If such missing data remains unaddressed, it may induce bias within the data distribution, adversely impacting the accuracy of model training and prediction. The objective of missing data processing is to maintain data integrity, thereby offering a more precise and comprehensive basis for ensuing data analysis and model training. In this study, the K-nearest neighbor (KNN) interpolation method was selected for processing missing data. KNN is an instance-based learning approach that leverages data similarities to predict missing values. KNN interpolation surpasses mean or median interpolation in efficiency as it captures the local structural information inherent in the data, yielding more accurate predictions.

Data discretization is another pivotal step in data preprocessing. It aims to convert continuous numerical data into discrete categorical data, thereby simplifying the computational process of the model and enhancing computational efficiency. DBNs are ideally suited to process discrete data, operating under the assumption that input data are discrete. Data discretization can diminish the influence of data noise to a certain degree and is more adept at capturing nonlinear relationships. For data discretization in this study, the K-means algorithm was employed, discretizing each variable into three status categories: low (L), medium (M), and high (H). The K-means algorithm, a commonly used clustering method, partitions the data into K clusters via an iterative process. This process minimizes the sum of the distances from each data point to the centroid of its respective cluster. During data discretization, each cluster is treated as a discrete category. The K-means based discretization method effectively accounts for the data distribution and can automatically define the range of each category. It is well-suited to handle complex, non-uniformly distributed data, thereby exhibiting excellent performance.

Structure learning in DBNs forms the nucleus of the DBN modeling process, dictating the basic architecture of the model. DBN structures are trained to infer causality from data, typically employing either score-based or constraint-based methodologies. In this study, we opted for the score-based approach, utilizing the Bayesian Information Criterion (BIC) scoring method in conjunction with the hill-climbing algorithm for learning the DBN structure. The fundamental principle of the BIC scoring method involves scoring potential network structures. This scoring system strikes a balance between the goodness of fit and the model dimension to avert overfitting, thereby facilitating a faster understanding of the real network. BIC is represented by

(1) $$S_{BIC}\left(G,\;D\right)=S_{LL}\left(G,\;D\right)-\frac{logN}{2}\cdot F,$$

where $S_{BIC}\left(G,\;D\right)$ is the BIC score of the DBN structure G and data D, $S_{LL}$ is the log likelihood score, N is the sample size, and F is the complexity penalty item. However, pinpointing the highest scoring network structure is a computationally challenging task, as the number of possible network structures escalates exponentially with the count of variables. The hill-climbing algorithm, a local search algorithm, seeks a state with a superior score in the vicinity of the current state, subsequently relocating the current state to this newly found state. This process is repeated until a better state cannot be identified. Although the hill-climbing algorithm may encounter local optima, it generally delivers satisfactory results in practical applications.

Parameter learning in DBN represents a critical task within the DBN modeling process. This study opted for the Bayesian estimation method and the Bayesian Dirichlet Equivalent Uniform Prior (BDeu) as the prior distribution to learn the parameters of the DBN. The Bayesian estimation approach incorporates prior knowledge, and despite its higher computational complexity, it effectively circumvents the issue of overfitting, thereby bolstering the stability and robustness of the model. BDeu priors are particularly beneficial for discrete variables, operating under the assumption that all parameter values are equally probable. As this prior does not rely on specific data, it proves suitable for scenarios characterized by limited data or substantial data missingness. The basis of Bayesian estimation method is Bayesian theorem, and its calculation formula is:

(2) $$P\left(\theta |X\right)=\frac{P(X│\theta )P(\theta )}{P(X)}$$

### 2.4. Dynamic Bayesian Network Evaluation and Validation

While modeling the dynamic causality of air quality in Hong Kong and Macao, the complexity is underscored by the characteristics of time series, multi-dimensionality, instability, and uncertainty. Accordingly, this study employed specific evaluation and validation techniques for the DBN, including predictive performance and sensitivity analysis.

In evaluating predictive performance, this study employed a multi-faceted and multi-tiered evaluation strategy to thoroughly assess the model’s performance. Firstly, data from 2016 to 2021 served as the training dataset, while the 2022 data functioned as the testing dataset, thereby providing a reliable estimate of the model’s generalization capability. This approach not only allowed for full data utilization, but it also ensured the model’s predictive performance on unfamiliar data. Secondly, a confusion matrix was used to elucidate the model’s predictive performance across different classes. The confusion matrix mirrored the model’s performance concerning true positives, true negatives, false positives, and false negatives, and was instrumental in evaluating key performance indicators such as precision, recall, specificity, and F1 scores of the model.

In conducting sensitivity analysis, this study adopted a comprehensive and systematic approach to quantify and comprehend the effect of changes in input parameters on the model’s output, and to identify the parameters that exert a decisive influence on model predictions. Initially, mutual information value analysis was employed to gauge the interdependence between two random variables, thereby quantifying the degree of correlation between the input and output of a DBN. Subsequently, the results from the mutual information value analysis were used to delve into the parameters exhibiting higher sensitivity, facilitating an understanding of these parameters’ influence mechanisms.

## 3. Results and Discussion

Considering the unique characteristics of the atmospheric environment in Hong Kong and Macao, this study incorporated eight meteorological variables specific to both regions, alongside four air quality variables for each territory, and an additional variable relevant to both regions. The DBN modeling process was implemented in several stages. Initial data cleaning was performed using the KNN interpolation technique for managing missing data, followed by the K-means algorithm for data discretization. Once preprocessed, the data were employed to construct the DBN structure via the BIC scoring method and the hill-climbing algorithm. Bayesian estimation and BDeu were adopted as the prior distribution for the learning of DBN parameters. As a result, two distinct DBNs were established for Hong Kong and Macao air quality, respectively, each comprising 13 variables. These DBNs were further divided into two sub-networks: the meteorological and air quality sub-networks. These sub-networks interact dynamically, reflecting the multifaceted linkage effects present within the atmospheric environments of Hong Kong and Macao.

In accordance with the unique atmospheric characteristics of Hong Kong and Macao, this study formulated a DBN model to assess air quality. It incorporated targeted variables such as PM_10_, PM_2.5_, CO, and NO_2_ for both regions, designating these as research nodes. In Hong Kong, PM_10_ concentrations were primarily influenced by MT and LT, while PM_2.5_ levels were chiefly determined by MT and Td. CO concentrations were significantly affected by MT and LT, whereas NO_2_ levels largely depended on MT and SSI. Conversely, in Macao, PM_10_ concentration was predominantly influenced by MT and RH. The concentration of PM_2.5_ was largely determined by MT and Td, while CO levels were primarily influenced by MT and Td. Lastly, O_3_ concentration was primarily affected by MT and SSI.

**Figure 3:**
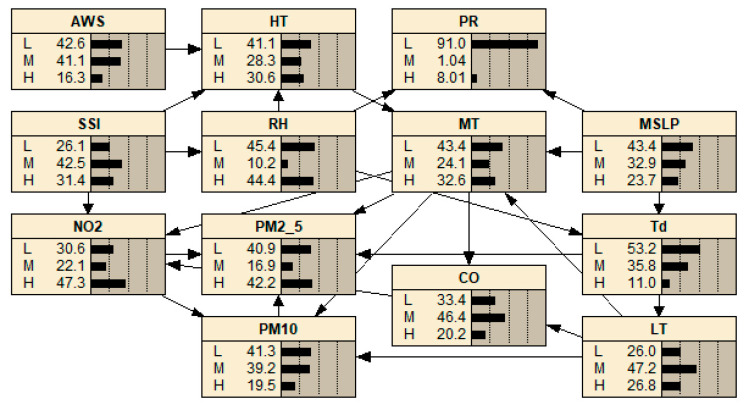
The dynamic causal model of air quality in Hong Kong.

**Figure 4:**
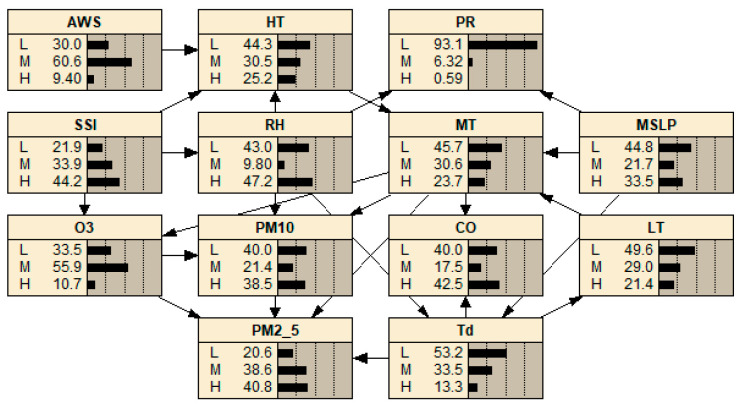
The dynamic causal model of air quality in Macao.

The interplay between atmospheric temperature and the generation and diffusion of airborne particulates and gases such as PM_10_, PM_2.5_, CO, NO_2_, and O_3_ was notable. High temperatures can expedite chemical reactions, thereby enhancing the formation of these substances. Conversely, lower temperatures can inhibit these reactions, subsequently diminishing the generation of these pollutants. Furthermore, elevated temperatures augment atmospheric convection, which, in turn, stimulates the diffusion of PM_10_, PM_2.5_, CO, and NO_2_. On the other hand, a decrease in temperature fosters air stability, thus inhibiting the diffusion of the aforementioned pollutants. Atmospheric humidity also significantly influences the creation and metamorphosis of PM_10_. With higher humidity, PM_10_ particles grow in size and their sedimentation rate escalates. Humidity, by mediating atmospheric chemical reactions, consequently impacts the concentration of PM_10_. Td, a measure of air humidity, is a critical factor in the production and transformation of PM_2.5_ and CO. Should the ambient temperature dip below the dew point, water vapor in the air condenses on PM_2.5_ surfaces, enlarging the particles and increasing their sedimentation rate. A surge in humidity stabilizes the air and constrains the dispersion of CO, and by governing atmospheric chemical reactions, it influences the concentration of PM_2.5_ and CO. Additionally, the duration of sunlight affects the concentration of NO_2_ and O_3_, as it influences photochemical reactions in the atmosphere. Extended sunshine can deplete NO_2_ concentration while boosting O_3_ levels. In contrast, a reduction in daylight hours could result in an increase in NO_2_ levels and a concurrent decrease in O_3_ concentration. The dynamic causal model of air quality in Hong Kong is shown in Figure 3. The dynamic causal model of air quality in Macao is shown in Figure 4. Figure 3 and Figure 4 illustrate the internal structure of a DBN, represented in terms of time slices.

The evaluation and validation of a DBN encompass both predictive performance and sensitivity analysis. During the assessment of predictive performance, the data from 2016 to 2021 were initially used as the training dataset, while the data from 2022 served as the testing dataset. This strategy ensured a dependable estimation of the model’s ability to generalize. Following this, a confusion matrix was employed to shed light on the model’s predictive performance across diverse classes. For sensitivity analysis, mutual information value analysis was first applied to measure the interdependence between pairs of variables, thereby quantifying their degree of association. The results of this mutual information value analysis were then used to delve into a detailed examination of the parameters demonstrating high sensitivity, with the objective of gaining a clear comprehension of these parameters’ influence mechanisms.

**Figure 5:**
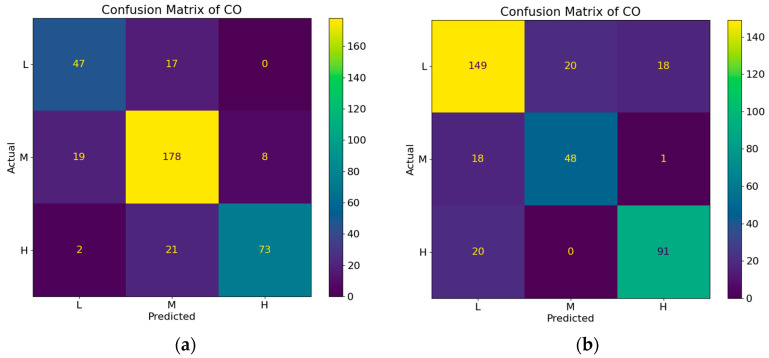
(**a**) The confusion matrix of CO predicted by the dynamic causal model of air quality in Hong Kong; (**b**) the confusion matrix of CO predicted by the dynamic causal model of air quality in Macao.

**Figure 6:**
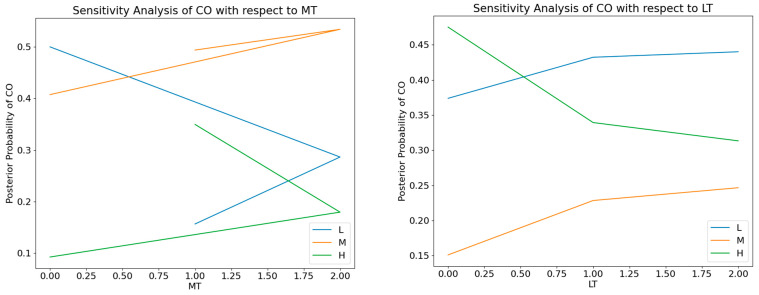

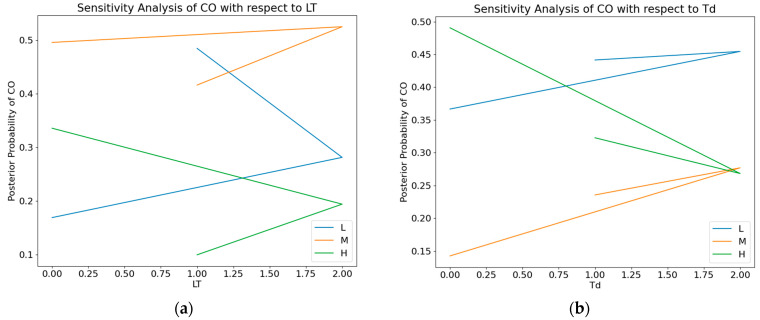
(**a**) The sensitivity analysis of CO predicted by the dynamic causal model of air quality in Hong Kong; (**b**) the sensitivity analysis of CO predicted by the dynamic causal model of air quality in Macao.

Considering the unique atmospheric environmental traits of Hong Kong and Macao, this study established a DBN model for air quality, selecting target variables PM_10_, PM_2.5_, CO, NO_2_, and O_3_ as the research nodes. Confusion matrices were constructed, classification reports were generated, and sensitivity analyses were conducted. The dynamic causal model performed well overall for assessing air quality in Hong Kong and Macao. Specifically, the Hong Kong Central Roadside Monitoring Station and Causeway Bay Roadside Monitoring Station predicted the accuracy of PM_2.5_ as 0.88, while the Macao Taipa General Station and Coloane General Station predicted the accuracy of PM_10_ as 0.86, respectively showcasing the best results. Notably, the weighted average of individual target variables exceeded the macro average, suggesting that the model performed more efficiently in categories with substantial sample sizes. In the Hong Kong air quality dynamic causal model, the variables exhibiting the highest correlation with CO were MT and LT. Sensitivity analysis graphs presented horizontal broken lines, speculated to arise due to non-linearity or randomness within the DBN, suggesting a complex relationship between CO and MT and between CO and LT. Conversely, in the Macao air quality dynamic causal model, the variables demonstrating the highest correlation with CO were LT and Td. The sensitivity analysis diagram of CO and LT generally exhibited a linear shape, indicating a relatively straightforward relationship between CO and LT. The confusion matrix of CO predicted by the dynamic causal model of air quality in Hong Kong and Macao is shown in Figure 5. The sensitivity analysis of CO predicted by the dynamic causal model of air quality in Hong Kong and Macao is shown in Figure 6. The classification report of the dynamic causal model of air quality in Hong Kong is shown in Table 2. The classification report of the dynamic causal model of air quality in Macao is shown in Table 3.

## 4. Conclusions

This study’s future research directions and potential applications are as follows: (1) In-depth research on data and models entails several pivotal approaches. Firstly, researchers explored a range of data preprocessing methods, assessing the impact of data cleaning, conversion, and feature selection on the predictive accuracy of the DBN model. Secondly, various DBN structure learning and parameter learning methods were implemented to elevate the model’s overall performance. Lastly, the intricacies of complex nonlinear relationships were delved into, aiming to offer a more precise and nuanced prediction. (2) In this research, factors beyond meteorology were explored to understand their influence on air quality. For the dynamic causal model focused on Hong Kong and Macao, various non-meteorological factors were incorporated. These included emissions from traffic, ships, and industries; energy production and consumption; regional collaboration and transboundary pollution; regulatory policies; building construction; residential emissions; tourism; urbanization and land use shifts; waste disposal and burning; as well as underlying socio-economic factors. (3) In research comparing other geographical regions. Firstly, select cities or regions with geographical and climatic characteristics similar to Hong Kong and Macao. Assess their similarities and differences in a range of influential factors, aiming to understand how these variables manifest in diverse geographical settings. Secondly, take into account unique regional influences such as population density and economic development. Refine and broaden the DBN accordingly to delve deeper into the decision-making and atmospheric environment management in these varying regions. Lastly, establish a continuous monitoring and feedback mechanism, ensuring periodic evaluation of model management strategies to cater to the needs of other geographical regions. Each of these strategies has the potential to contribute significantly to the field and warrants further research.

This study offers three key contributions: (1) It applied an interpretable DBN to construct a dynamic causal model of air quality in Hong Kong and Macao, amidst complex, unstable, multi-dimensional, and uncertain factors over time. (2) It investigated the dynamic interaction between meteorology and air quality sub-networks, and both qualitatively and quantitatively identified, evaluated, and understood the causal relationships between air pollutants and their determinants. (3) It facilitated an online collaborative forecast of air pollutant concentrations, enabling pollution warnings. The findings corroborate the potency of a DBN-based dynamic causal model in explaining and managing complex atmospheric environmental systems. Such a model has significant referential value not only for decision-making and atmospheric environmental management in Hong Kong and Macao but also for neighboring cities and regions with geographical environments analogous to these areas.

## Figures and Tables

**Table 1:** Data type and source, variable selection and description.

Data Type	Source	Variables	Description
Meteorological data for Hong Kong and Macao	Hong Kong Observatory Macao Meteorological and Geophysical Bureau	MSLP	mean sea level pressure (hPa)
		HT	highest temperature (°C)
		MT	mean temperature (°C)
		LT	lowest temperature (°C)
		Td	dew point temperature (°C)
		RH	relative humidity (%)
		AWS	average wind speed (km/h)
		PR	precipitation (mm)
Air quality data for Hong Kong	Hong Kong Environmental Protection Department (the Central Roadside Monitoring Station, the Yuen Long General Monitoring Station, and the Causeway Bay Roadside Monitoring Station)	PM_10_	coarse particulate matter (μg/m^3^)
		PM_2.5_	fine particulate matter (μg/m^3^)
		CO	carbon monoxide (mg/m^3^)
		NO_2_	nitrogen dioxide (μg/m^3^)
Air quality data for Macao	Macao Environmental Protection Bureau (the Macao High Density Residential Area Station, the Taipa General Station, and the Coloane General Station)	PM_10_	coarse particulate matter (μg/m^3^)
		PM_2.5_	fine particulate matter (μg/m^3^)
		CO	carbon monoxide (mg/m^3^)
		O_3_	ozone (μg/m^3^)
Other data for Hong Kong and Macao	Hong Kong Observatory Macao Meteorological and Geophysical Bureau	SSI	sunshine duration (h)

**Table 2:** The classification report of the dynamic causal model of air quality in Hong Kong.

Hongkong	Target	BIAS	Accuracy	Macro Avg			Weighted Avg		
				Precision	Recall	F1-Score	Precision	Recall	F1-Score
the Central Roadside Monitoring Station	PM_10_	0.01	0.85	0.77	0.90	0.82	0.87	0.85	0.86
	PM_2.5_	−0.03	0.88	0.91	0.78	0.82	0.89	0.88	0.88
	CO	0.26	0.74	0.79	0.81	0.79	0.79	0.74	0.75
	NO_2_	0.10	0.78	0.76	0.72	0.78	0.80	0.78	0.78
the Yuen Long General Monitoring Station	PM_10_	0.15	0.77	0.73	0.78	0.76	0.82	0.77	0.79
	PM_2.5_	−0.04	0.82	0.85	0.73	0.77	0.83	0.82	0.81
	CO	−0.05	0.82	0.81	0.79	0.79	0.82	0.82	0.82
	NO_2_	−0.09	0.77	0.70	0.58	0.70	0.78	0.77	0.76
the Causeway Bay Roadside Monitoring Station	PM_10_	0.00	0.86	0.82	0.90	0.85	0.87	0.86	0.86
	PM_2.5_	0.19	0.88	0.87	0.86	0.85	0.91	0.88	0.89
	CO	0.09	0.78	0.75	0.74	0.75	0.78	0.78	0.76
	NO_2_	−0.14	0.79	0.78	0.70	0.79	0.81	0.79	0.80

**Table 3:** The classification report of the dynamic causal model of air quality in Macao.

Macao	Target	BIAS	Accuracy	Macro Avg			Weighted Avg		
				Precision	Recall	F1-Score	Precision	Recall	F1-Score
the Macao High Density Residential Area Station	PM_10_	−0.06	0.84	0.79	0.84	0.81	0.85	0.84	0.84
	PM_2.5_	0.25	0.79	0.79	0.72	0.74	0.83	0.79	0.80
	CO	0.00	0.85	0.82	0.82	0.82	0.85	0.85	0.85
	O_3_	−0.07	0.79	0.81	0.78	0.79	0.80	0.79	0.79
the Taipa General Station	PM_10_	−0.05	0.86	0.83	0.77	0.79	0.86	0.86	0.86
	PM_2.5_	−0.04	0.85	0.81	0.81	0.81	0.85	0.85	0.85
	CO	0.00	0.79	0.78	0.78	0.78	0.79	0.79	0.79
	O_3_	0.02	0.78	0.77	0.75	0.76	0.78	0.78	0.78
the Coloane General Station	PM_10_	−0.06	0.86	0.79	0.80	0.79	0.86	0.86	0.86
	PM_2.5_	0.22	0.83	0.76	0.71	0.71	0.86	0.83	0.84
	CO	−0.06	0.79	0.80	0.79	0.79	0.80	0.79	0.79
	O_3_	0.05	0.73	0.76	0.72	0.73	0.75	0.73	0.74

## Data Availability

The data for Hong Kong were sourced from the Hong Kong Observatory: https://www.weather.gov.hk/en/cis/dailyExtract.htm?y=2016&m=1 (accessed on 1 May 2023) and the Hong Kong Environmental Protection Department: https://cd.epic.epd.gov.hk/EPICDI/air/station/?lang=en (accessed on 1 May 2023). The data for Macao were sourced from the Macao Meteorological and Geophysical Bureau: https://www.smg.gov.mo/en/subpage/345/embed-path/p/query-weather-e_panel (accessed on 1 May 2023) and the Macao Environmental Protection Bureau: https://www.dspa.gov.mo/envdata.aspx (accessed on 1 May 2023).
